# Faunistic Inventory of Spheciformes Wasps at Three Protected Areas in Portugal

**DOI:** 10.1673/031.013.11301

**Published:** 2013-10-26

**Authors:** L. C. Vieira, N. G. Oliveira, C. C. Brewster, S. F. Gayubo

**Affiliations:** 1 Department of Entomology, Virginia Tech, Blacksburg, VA 24061-0319, USA; 2 CIGEST — Business Management R & D Centre, Sustainability area, Instituto Superior de Gestão, 1750-306 Lisboa, Portugal; 3 Unidad de Zoología, Departamento de Biología Animal, Facultad de Biología, Universidad de Salamanca, 37071 Salamanca, Spain; 4 Department of Forest Protection and Entomology, Faculty of Forestry and Wood Sciences, Czech University of Life Sciences Prague, Kamycka 1176, CZ-165 21 Prague, Czech Republic

**Keywords:** Douro International Natural Park, faunistic catalogue, Hymenoptera, Paúl do Boquilobo Nature Reserve, Serras de Aire e Candeeiros Natural Park, species richness

## Abstract

The importance of considering insects in the protection of biodiversity has been recently recognized. However, despite the importance of Spheciformes wasps (Hymenoptera: Ampulicidae, Sphecidae and Crabronidae) in natural ecosystems and their potential as bioindicators, the Spheciformes communities in Portugal (part of the European biodiversity hotspot) have rarely been studied, and data for Portuguese protected areas are scarce. The Spheciformes wasp communities at 3 protected areas in Portugal, Douro International Natural Park, Serras de Aire e Candeeiros Natural Park, and Paúl do Boquilobo Nature Reserve, were studied in 2000 and 2001. During the study, 134 species of Spheciformes belonging to 3 families, Ampulicidae, Sphecidae, and Crabronidae, were identified. The species collected constituted nearly 1/3 of the species known in the Iberian Peninsula, 42 were new records for Portugal. Additionally, several specimens of 6 potentially new species were collected. Douro International Natural Park had the highest species richness, followed by Serras de Aire e Candeeiros Natural Park and Paúl do Boquilobo Nature Reserve. All the protected areas studied had species that were found exclusively at an individual protected area and species that were found to be new records for Portugal. Based on the literature review of the geographic distribution, nidification types, and prey orders, it was found that most species collected had a Euroasiatic or Mediterranean distribution, species with fossorial habits predominated, and the orders/suborders of insects preyed upon by most species were Diptera, Orthoptera, Sternorrhyncha, and Auchenorrhyncha. This study underscores the importance of including the protected areas studied in the conservation of Spheciformes diversity and also suggests that insect diversity should be studied separately, as it does not necessarily follow the same patterns as other, more studied, groups.

## Introduction

Biodiversity is one of the most important elements in national and international legislation for the selection of conservation areas. Birds Directive, Habitats Directive, and the IUCN Red List of Threatened Species, for example, specifically include lists of species of special interest for conservation. However, despite its importance, knowledge of the biodiversity of most protected areas is limited, focusing primarily on vertebrates. The most noticeable gap in knowledge is with respect to arthropod diversity. Despite being the most diverse animal group, representation of arthropods in biodiversity inventories and listings has been and remains minimal (New 2012). Knowledge of the entomological fauna in Portugal, for example, is very limited in general, but is especially scarce or absent for Hymenoptera (Kuhlmann 1996), such as Spheciformes (Hymenoptera: Ampulicidae, Sphecidae and Crabronidae). In order to manage and evaluate the effectiveness of protected areas at preserving biodiversity, it is important to have a comprehensive knowledge on the diversity of species under protection.

The variety of roles arthropods play in ecosystems—as herbivores, predators, decomposers, parasites, pollinators, and seed dispersers—require that any evaluation of ecosystem functioning should necessarily include arthropods (Maleque et al. 2006). Spheciformes wasps play an important role in ecosystems as predators. Because previous studies have shown the potential of Spheciformes as bioindicators (Gayubo et al. 2005; Vieira et al. 2011), an inventory of this group with taxonomic, ecological, and biogeographic data collected systematically (Strumia et al. 2002) could be useful for monitoring biodiversity in protected areas. Furthermore, considering the current “biodiversity crisis” (Clausnitzer et al. 2009; Lawrence and Wright 2009; Peh 2011), studies on lesser-known groups, such as Spheciformes, are especially valuable for future and current conservation efforts. Biodiversity inventories in Portugal are not only relevant at a national level but also from an international perspective, as Portugal is part of the European biodiversity hotspot (Myers et al. 2000).

The aim of this study was to contribute to the knowledge of Spheciformes wasps in Portugal. A listing and quantification of the Spheciformes were made for 3protected areas, namely Douro International Natural Park (DINP), Serras de Aire e Candeeiros Natural Park (SACNP), and Paúl do Boquilobo Nature Reserve (PBNR). The abundance, diversity, geographic distribution, and some aspects of the basic biology, such as nidification types and potential prey, were also determined. The knowledge gathered in this study partially fills the gap of information on Spheciformes in Portugal (part of the European biodiversity hotspot), complements the biodiversity lists for these areas, provides relevant information for ecosystem functioning recognizing the role of this group as population regulators (predators) for other groups, and provides a baseline for the future monitoring of management in the protected areas.

**Figure 1.**
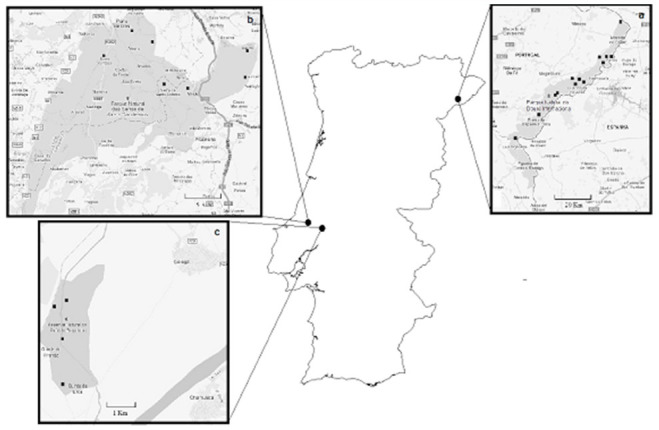
Geographic location and the limits of the Douro International Natural Park (a), Serras de Aire e Candeeiros Natural Park (b), and Paúl do Boquilobo Nature Reserve (c), Portugal. Points on the map of each area show the distribution of sampling sites. High quality figures are available online.

## Materials and Methods

### Study areas

The study was conducted at 3 Portuguese protected areas (Figure 1), DINP, SACNP, and PBNR.

The DINP is located on a 122 km border section of the Douro River (41.277806° N, 6.635742 ° W) (Figure 1a). It covers a total area of 85,150 ha (Anonymous 2001b). The border section of Douro River makes the transition between the medium and the lower river basin through a steep longitudinal slope. The northern part of DINP is characterized by an extensive plateau with altitudes ranging from 700 to 800 m a.s.l. The valley is tightly set between granitic steep slopes. Going south, the valley is more open, and the granitic steep slopes remain but there is an open plateau at the bottom (Anonymous 2001b). The climate in the northern part is subcontinentalmediterranean, with a wide thermal range, very cold winters, and very hot and dry summers. The southern part has a microclimate similar to that of the Douro Wine Region, which is characterized by low precipitation and mild winter temperatures (Anonymous 2001b). DINP was established in 1998; its international value has been recognized under the Habitats (1997) and Birds (1999) Directives.

The SACNP is located in the Midwest area of Portugal (39.518344° N, 8.788376° W) (Figure 1b). It has a total area of 39,900 ha (Anonymous 2001a). Most of SACNP is included on the Estremadura's Limestone Massif. Morphologically, the Estremadura's Limestone Massif can be differentiated into 4 elevated sub-units, Candeeiros Mountain (west), Santo António (south central) and São Mamede (north) Plateaus, and Aire Mountain (east), separated by 3 great depressions, Mendiga, Minde-Mira Polje, and Alvados. While there is little surface freshwater, groundwater is abundant and is responsible for the multitude of karst formations in the area (Anonymous 2001a). The climate is atlanticmediterranean, characterized by high humidity levels, mild temperatures, and dry summers (Anonymous 2001a). SACNP was established in 1979 and its international value has also been recognized under the Habitats Directive (1997) and the Ramsar Convention (2006).

The PBNR is also located in the Midwest area of Portugal in the Almonda River basin (39.347839° N, 8.528481° W) (Figure 1c). It occupies an area of 554 ha (Anonymous 2001c) and covers the transition between fluvial terraces and alluvial flatlands of the Almond River. The alluvial flatlands have several riparian galleries that follow a complex network of water lines. The various riparian galleries have configurations and characteristics that reflect the history of this area, of which some have been restored in the full protection area (196 ha) and others show the influence of current or previous agricultural exploration in the area (Anonymous 2001c). Because PBNR is located in the same region as SACNP, their climatic characteristics are similar (Anonymous 2001c). The international value of PBNR has also been recognized under The Man and the Biosphere Program-UNESCO (1981) and the Ramsar Convention (1986).

### Specimen collection

The sampling sites at each protected area were selected in an effort to cover the majority of the habitats represented. Fourteen sites were selected at DINP, 7 at SACNP and 4 at PBNR (Supplemental Table 1).

Because of the distances between the protected areas and the large number of sites involved, it was not feasible to sample all sites during the same year and with the same frequency. DINP was sampled in 2001, and SACNP and PBNR were sampled in 2002. The sampling effort was classified as high, medium, or low for each site. A high sampling effort consisted of biweekly continuous sampling from April to September, a medium sampling effort consisted of biweekly continuous sampling from June to August, and a low sampling effort consisted of only 1 to 3 sampling periods during May and June. Two sampling methods were used, namely Malaise traps and flight interception traps with blue and yellow trays. At each sampling site, 1 Malaise and 1 flight interception trap were used. Malaise traps were made of a fine mesh, with black sides and central panels and a white top, following the design of Townes (1972). The flight interception traps were a modified version of the Masner and Goulet (1981) model consisting of a 2.5 m × 1 m panel of fine black mesh soaked with insecticide, and yellow and blue collection trays filled with water, detergent, and thymol. The 2 sampling methods were used in combination in order to obtain a more representative sample of the Spheciformes communities in each area (Noyes 1989; Campos et al. 2000).

All Spheciformes specimens collected were preserved in ethanol before being mounted for identification to the species level (Bitsch and Leclercq 1993; Bitsch et al. 1997; Prentice 1998; Brothers 1999; Melo 1999; Bitsch et al. 2001).

The geographic distribution (Pulawski 2011), nidification type, and prey orders consumed (Gayubo 1980; Gayubo et al. 2004; Baños- Picón et al. 2006) were determined for all species. The percentage of species previously found for the Iberian Peninsula was calculated (Gayubo et al. 2008).

### Statistical Analyses

Data on the geographic distribution, nidification type, and prey orders consumed were summarized for each of the protected areas. The Renkonen index (Renkonen 1938; Krebs 1998) was also calculated to provide a measure of percent similarity among the 3 study areas with respect to the 3 variables derived for the species collected.

A methodological problem of all faunistic inventories is the impracticability of registering all of the species in a given area, which is necessary for determining total species richness. The nonparametric estimators abundance-based coverage, Chao1, Chao2, first-order Jacknife, second-order Jacknife, and Bootstrap were therefore used to estimate Spheciformes total species richness at each of the protected areas, taking into consideration the variation in sampling effort, sampling methods, and size of the area (Hortal et al. 2006). Species accumulation-based total richness estimates are more reliable than traditional diversity indexes (which are affected by sampling pattern and size) for comparing faunistic studies in different areas (Jiménez-Valverde and Hortal 2003). As such, this approach was also used for the estimation of total species richness at the 3 protected areas. For the estimation based on the species accumulation curve, the number of samples was used as the sampling effort unit (17 samples maximum), and data input was randomized 1,000 times to obtain an optimized accumulation curve (Jiménez-Valverde and Hortal 2003). The values obtained were then fitted to the Clench equation:

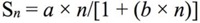
where S_*n*_ is the number of species, *a* is the rate of increase of new species at the start of sampling, *b* is a parameter related to the shape of the curve, and *n* is the sampling effort. The model equation was fitted to the data using the Simplex and Quasi-Newton Method. The total species richness was then determined by calculating the horizontal asymptote of the curve:

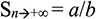
Two methods were used to evaluate the completeness of the inventories: (1) calculation of the proportion of species richness observed (*S_obs_*) in relation to the total richness predicted by the nonparametric estimators (*S_est_*), and (2) determination of the slope of the accumulation curve:

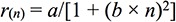
Statistical analyses were performed in EstimateS 8.2.0 (Colwell 2005) and Statistica (StatSoft 2007).

## Results

A total of 2,970 specimens were collected during the study. The specimens represented 134 species belonging to 46 genera, 17 tribes, and 3 families (Supplemental Table 2). These constituted 29% of the species and 64% of the genera known from the Iberian Peninsula. Although all species collected have been recorded previously for the Iberian Peninsula, 42 species (31%) are new records for Portugal.

### Species composition

**Figure 2.**
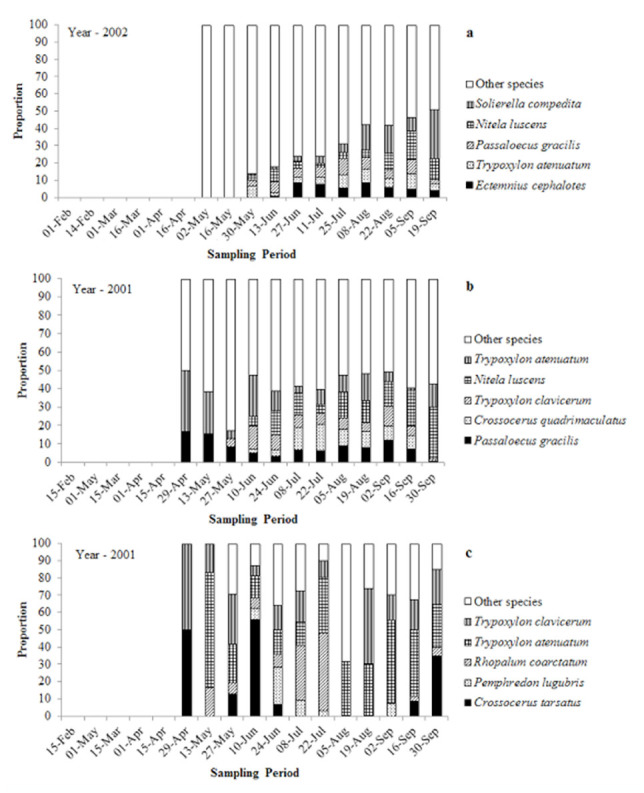
Relative percentage of the 5 most abundant species of Spheciformes wasps collected during each of the sampling periods at Douro International Natural Park (a), Serras de Aire e Candeeiros Natural Park (b), and Paúl do Boquilobo Nature Reserve (c), Portugal. High quality figures are available online.

At DINP, 118 species and 5 morphospecies (potentially new species to science) belonging to 43 genera, 17 tribes, and 3 families, were collected. Thirty-five species were new records for Portugal, and 55 species, 4 morphospecies, and 11 genera were exclusive to DINP (Supplemental Table 2). The occurrence of the 5 most abundant species at DINP increased as the sampling season progressed to a maximum of 51% of the samples, with an average of 27% (Figure 2a).

Seventy species and 1 morphospecies belonging to 34 genera, 14 tribes, and 3 families were collected at SACNP. Twenty-one of the species were new records for Portugal, and 13 species, 1 morphospecies, and 1 genus were exclusive to SACNP (Supplemental Table 2). The occurrence of the 5 most abundant species at SACNP was consistent throughout the sampling season, averaging about 42% of the samples (Figure 2b).

At PBNR, 27 species and 1 morphospecies belonging to 17 genera, 11 tribes, and 3 families were collected. Nine species were new records for Portugal, and 3 species were exclusive to PBNR (Supplemental Table 2). The occurrence of the 5 most abundant species at PBNR was fairly high throughout the sampling season, with an average of 76% of the samples (Figure 2c).

#### Geographic distribution

Most species collected in all study areas had a Euroasiatic (38%) or Mediterranean (32%) distribution. Other species were distributed in Europe and Asia but also in North America (14%), Africa (10%), and South America (1%). The remaining species were endemic to the Iberian Peninsula (5%). The most dominant zoogeographical element at all natural areas was Euroasiatic. Neither Iberian nor South American species were collected at PBNR (Table 1). For all study areas, the species classified as North American, African, and South American were those that were not only distributed in Euroasia, but also in those continents.

The Renkonen index showed that the geographic distribution of species collected at DINP was more similar to those collected at SACNP (≈95%) than to those at PBNR (≈69%). The similarity between the geographic distribution of the species collected at SACNP and PBNR was ≈73%.

#### Nidification type

Most species collected in the study were fossorial (64%), making their nests on the ground. Other species were xylicolous (15%), which build their nests in soft core stems, hollow stems, or soft pieces of wood. Others either nested in pre-existing cavities (15%), had mixed behavior showing a combination of the nidification types described previously (2%), or were cleptoparasites (4%) that lay their eggs in other wasps nests (Supplemental Table 3). Species collected at DINP and SACNP were mostly fossorial. Cleptoparasites were not collected at SACNP. Unlike the other study areas, the species collected at PBNR were equally distributed among 3 nidification types, xylicolous, fossorial, or those that nested in pre-existing cavities. There were also less species with mixed behavior or that were cleptoparasites at PBNR (Table 2).

The Renkonen index showed that with respect to nidification type, the species collected at DINP were more similar to those collected at SACNP (≈94%) than to those at PBNR (≈64%). The similarity in species nidification type between SACNP and PBNR was ≈69%.

#### Prey consumed

**Figure 3.**
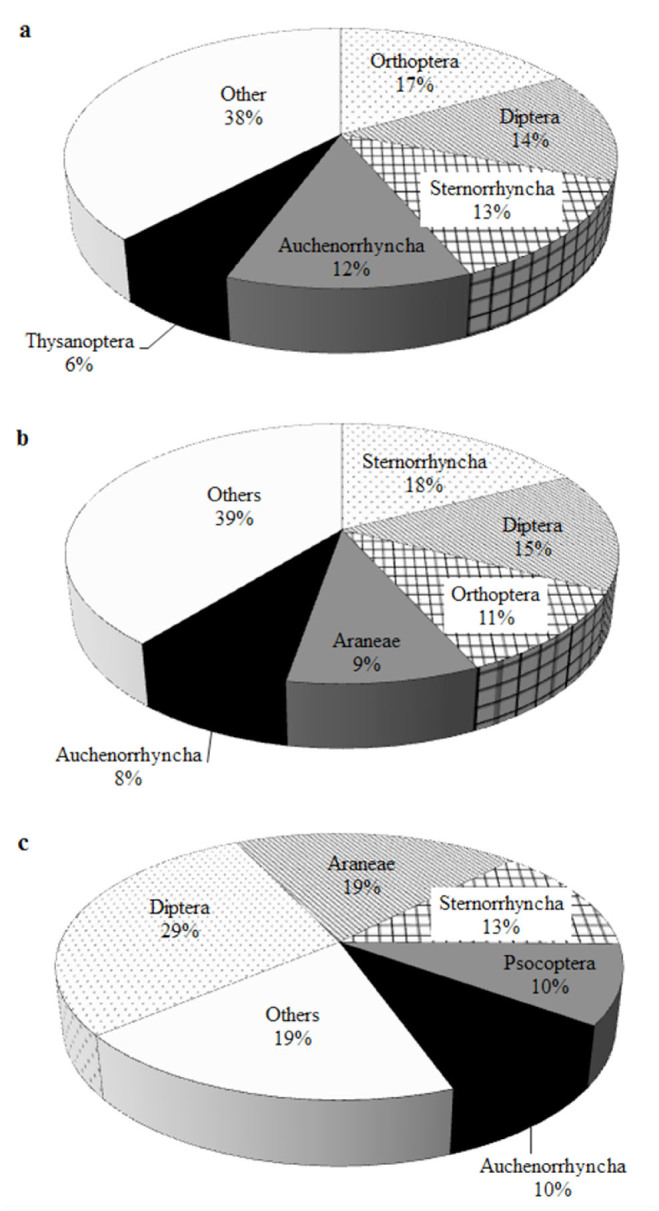
Relative percentage of the species of Spheciformes wasps capturing the 5 main prey orders/suborders of insects at Douro International Natural Park (a), Serras de Aire e Candeeiros Natural Park (b), and Paúl do Boquilobo Nature Reserve (c), Portugal. High quality figures are available online.

Most of the species collected preyed upon 4 main orders/suborders of insects: Diptera (16%), Orthoptera (16%), Sternorrhyncha (13%), and Auchenorrhyncha (13%). The remaining species preyed upon Heteroptera (8%) and 7 other orders (34%) (Supplemental Table 3). The 5 main orders preyed upon by the species varied according to the natural area (Figure 3a–c).

Based on the Renkonen index, the similarity in the prey species consumption preference by Spheciformes at DINP and SACNP was ≈85%. The similarity between DINP and PBNR was 63%, while the similarity between SACNP and PBNR was ≈68%.

#### Total species richness estimation

The highest species richness was observed and estimated for DINP, which varied between 139 and 184 species (Table 3). SACNP had lower observed and estimated species richness than DINP, but a higher species richness than PBNR; the estimated total species richness varied between 82 and 111 species (Table 3). The lowest species richness was observed and estimated for PBNR, as the estimated total species richness varied between 28 (similar to the number of species observed) and 42 species (Table 3).

#### Inventory completeness

**Figure 4.**
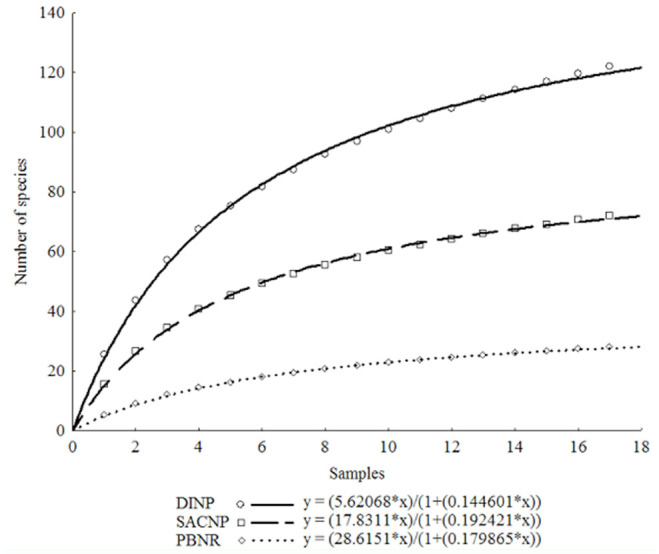
Species richness accumulation curves for the specimens of Spheciformes wasps collected at Douro International Natural Park (circles, solid line), Serras de Aire e Candeeiros Natural Park (squares, dashed line), and Paúl do Boquilobo Nature Reserve (rhombus, dotted line), Portugal. High quality figures are available online.

A good fit to Clench's model was obtained for the optimized accumulation curves for each study area (*R^2^* = 0.99, *p* < 0.01) (Figure 4). The percentage of observed species richness in relation to the estimated species richness collected varied between 67–87%, 69–88%, and 69–86% for DINP, SACNP, and PBNR, respectively (Table 3). The values for the final slope of the species accumulation curves were 2.76, 1.52, and 0.80 for DINP, SACNP, and PBNR, respectively.

### Discussion

While knowledge of vertebrate species diversity is extensive for the natural areas that were studied (Monteiro 1998; Brotas 2001; Pimenta and Correia 2001; Coelho 2007; Alexandrino et al. 2008; Alves et al. 2008; Raposo et al. 2008), there appears to be either a significant lack of or limited access to information on the entomological fauna. This was evident in this study by the large number of new records found for Portugal. This work not only generated records for Spheciformes at 3 protected areas in Portugal, but also added 42 species to the Portuguese inventory and potentially 6 new species for science.

The 3 natural areas studied harbor close to 1/3 of the Spheciformes species known in the Iberian Peninsula. DINP had the highest species richness, followed by SACNP and PBNR. This difference in species richness pattern is not consistent with the patterns for vertebrates groups (amphibians, birds, fish, and mammals) in these areas, with the exception of reptiles. However, the species richness patterns for the different vertebrate groups also were not consistent (Monteiro 1998; Coelho 2007; Raposo et al. 2008). The results of our study demonstrated the inadequacy of using vertebrate diversity as an indicator of Spheciformes diversity. This inconsistency between vertebrate and invertebrate diversity has been observed in other studies (Majer 1983; Burbidge et al. 1992; Oliver et al. 1998; Bennett et al. 2009). It should be noted that differences in the pattern of diversity (species richness) at the study areas could have resulted from the different number of sampling points (higher at DINP than at SACNP and PBNR) despite the fact that the number of sampling points was fairly proportional to the size of the protected area.

In addition to species richness, another important factor relevant for conservation is rarity (Rodrigues and Gaston 2002). All the protected areas studied had a number of species that were found exclusively at 1 of the areas and also species that represented new records for Portugal. Additionally, several specimens collected at DINP and SACNP potentially belong to 6 new species. Demographic rarities were also collected. These included the Iberian endemics *Bembecinus carpetanus* Mercet, *Bembecinus pulchellus* Mercet, *Stizus aestivalis* Mercet, *Nysson dusmeti* Mercet, *Nysson konowi* Mercet, and *Ammoplanus torresi* Gayubo collected at DINP, and *Entomognathus fortuitus* Kohl collected at SACNP.

Most species collected had a Euroasiatic or Mediterranean distribution. This pattern was fairly consistent in all areas studied. These results show the biogeographical importance of the protected areas studied as intersection areas, showing a strong representation of both Mediterranean and Euroasiatic biogeographic assemblages (González et al. 2009).

Considering all areas studied and the frequency of each nidification type, most species showed fossorial habits. The remaining species were mainly xylicolous or nested in preexisting cavities, and only a small proportion had mixed behavior or was cleptoparasite. The dominance of fossorial species was consistent with previous studies on other Iberian communities (González et al. 1998; Gayubo et al. 2000; Gayubo et al. 2004). Species at both DINP and SACNP followed this general pattern, while species at PBNR showed a very different pattern, with species that nested in pre-existing cavities, fossorial species, and xylicolous species being equally present. This discrepancy may be attributed to soil conditions (riparian gallery) that might make the PBNR area less suitable for fossorial species compared with the other 2 protected areas.

The orders most species preyed upon were Diptera, Orthoptera, Sternorrhyncha, and Auchenorrhyncha. Similar to the nidification habits, species at PBNR followed a different general pattern from species at DINP and SACNP. Again, this discrepancy might be related to specific characteristics of the PBNR area, which may be more favorable to the existence of different types of plant resources, as prey orders in all cases are mostly herbivores.

Because the percentage of species observed was generally > 70% of the species predicted, the inventory may be considered to be fairly complete. Jiménez-Valverde and Hortal (2001) referred to a cutoff value of < 0.1 for inventory completeness, but in this study the accumulation curves final slopes were always > 0.1. Despite this, the inventory can still be considered complete because the cutoff value in Jiménez-Valverde and Hortal (2001) was determined using the specimens or records as the sampling unit, while in this study the sampling periods were used.

This study provides new information on Spheciformes wasps in Portugal and specifically at 3 protected areas. The study also reaffirms the importance of including the protected areas in the conservation of Spheciformes diversity and calls attention to the fact that insect diversity does not necessarily follow the same patterns of vertebrates, which are more commonly used for the selection of protected areas (Oliver et al. 1998). Considering the importance of insects both in terms of diversity and ecosystem functions (Wilson 1987), the need for further studies focusing on Spheciformes wasps and other insect groups is clear.

**Table 1.**
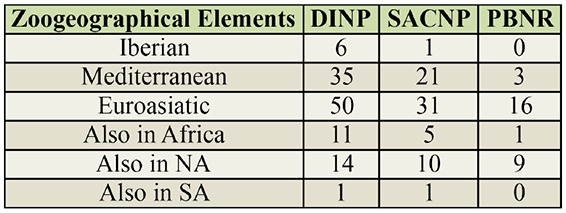
Number of Spheciformes wasps species representing the zoogeographical elements, Iberian, Mediterranean, Euroasiatic, African (also in Africa), North American (also in NA), and South American (also in SA) at Douro International Natural Park (DINP), Serras de Aire e Candeeiros Natural Park (SACNP), and Paúl do Boquilobo Nature Reserve (PBNR), Portugal. Species listed as also in NA, also in Af, and also in SA are present at these locations in addition to having a very wide distribution in Europe and Asia.

**Table 2.**
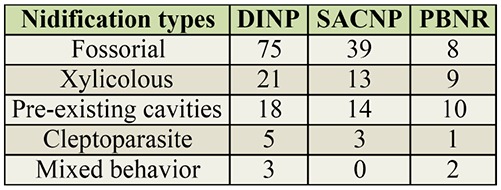
Number of Spheciformes wasps species with the nidification types, cleoptoparasite, fossorial, mixed behavior, pre-existing cavities, and xylicolous at Douro International Natural Park (DINP), Serras de Aire e Candeeiros Natural Park (SACNP), and Paúl do Boquilobo Nature Reserve (PBNR), Portugal.

**Table 3.**
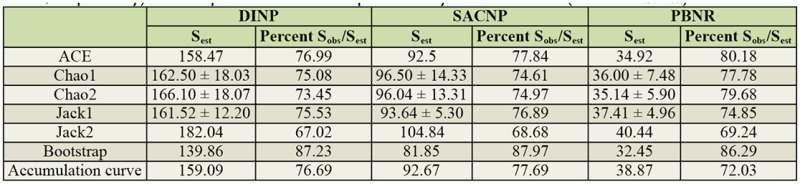
Estimated species richness (*S_est_*) (± SD where applicable) and the percentage of species observed (122, 72, and 28 at DINP, SACNP, and PBNR, respectively) with respect to the number predicted by each estimator (Percent *S_obs_*/*S_est_*).

**Supplemental Table 1.**
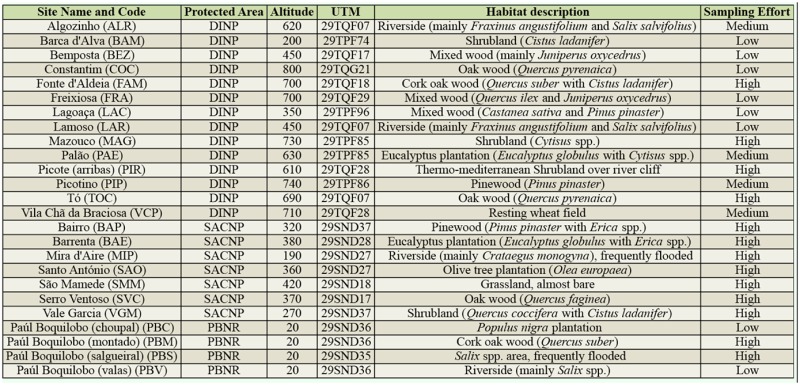
List of sampling sites with site name and code, protected area where the sample was taken, altitude (in meters), UTM coordinates, habitat description with the dominant vegetation indicated, and sampling effort.

**Supplemental Table 2.**
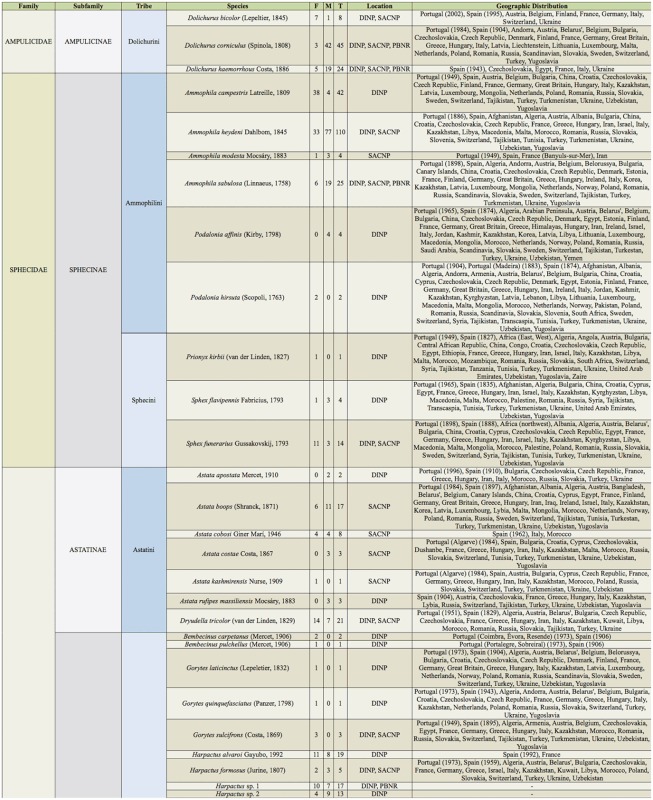
List of species collected separated into the corresponding families, subfamilies, and tribes; number of female specimens (F); number of male specimens (M); total number of specimens collected (T); location where specimens where collected (DINP: Douro International Natural Park, SACNP: Serras de Aire e Candeeiros Natural Park, PBNR: Paúl do Boquilobo Nature Reserve); and countries with previous records of each species, with indication of the year of the first published record in Portugal and Spain (geographic distribution).

**Continued.**
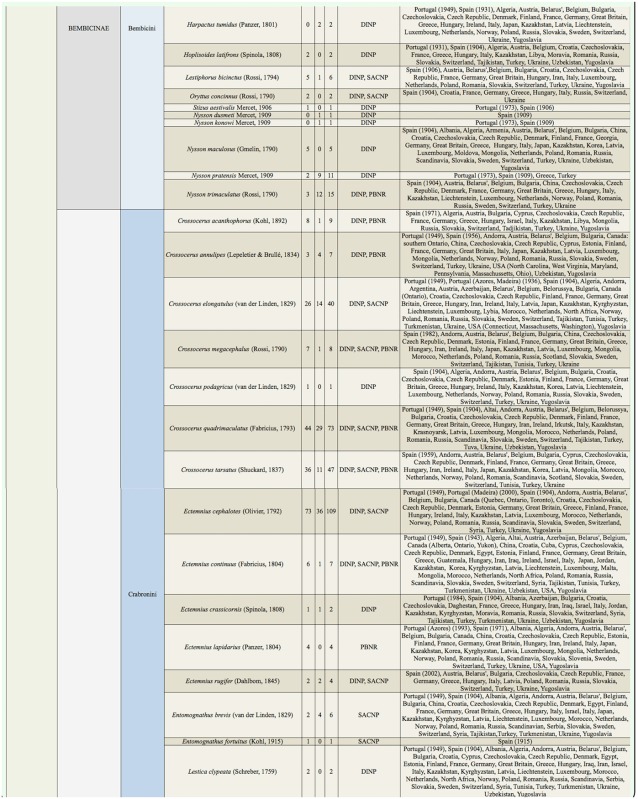

**Continued.**
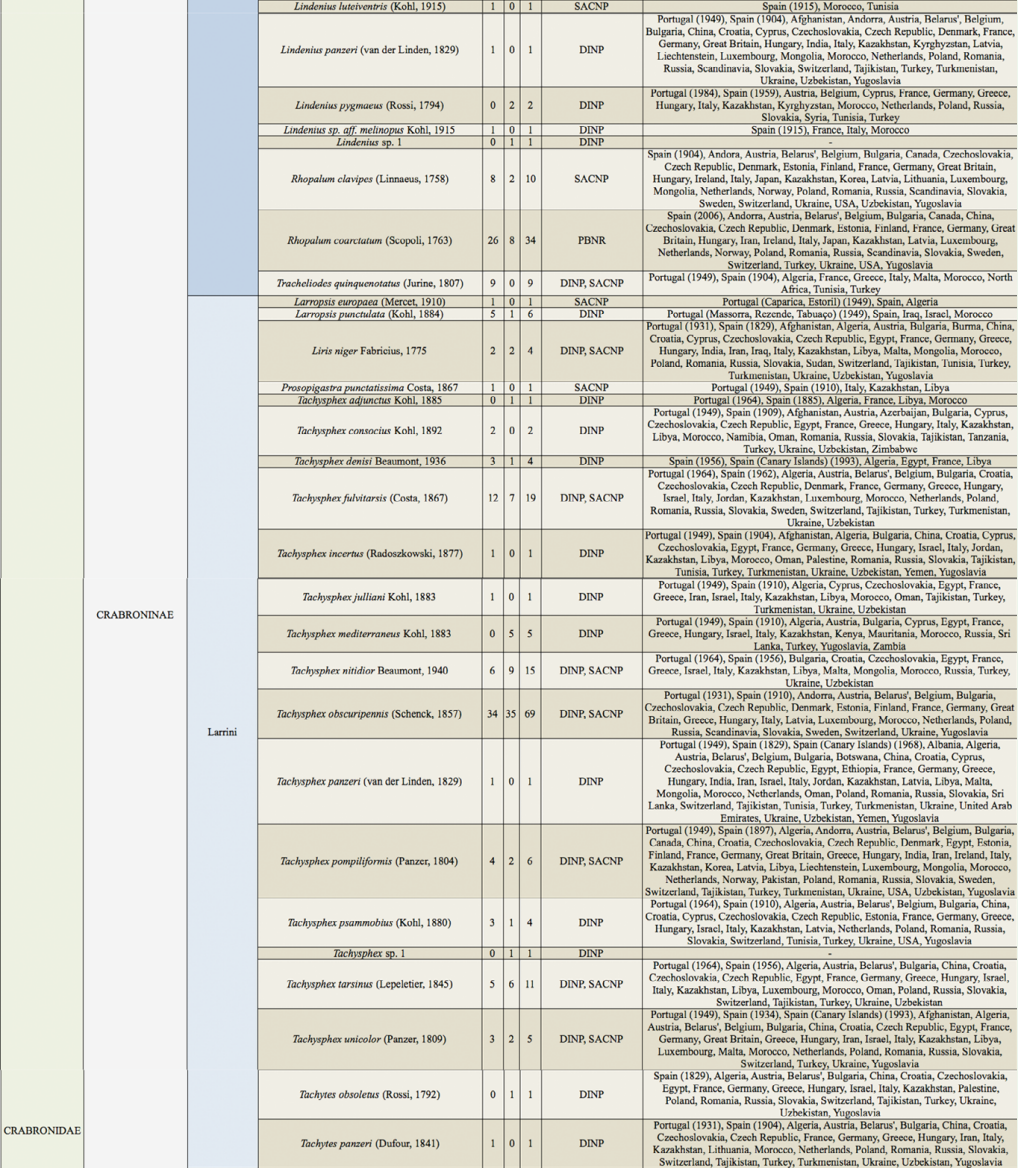

**Continued.**
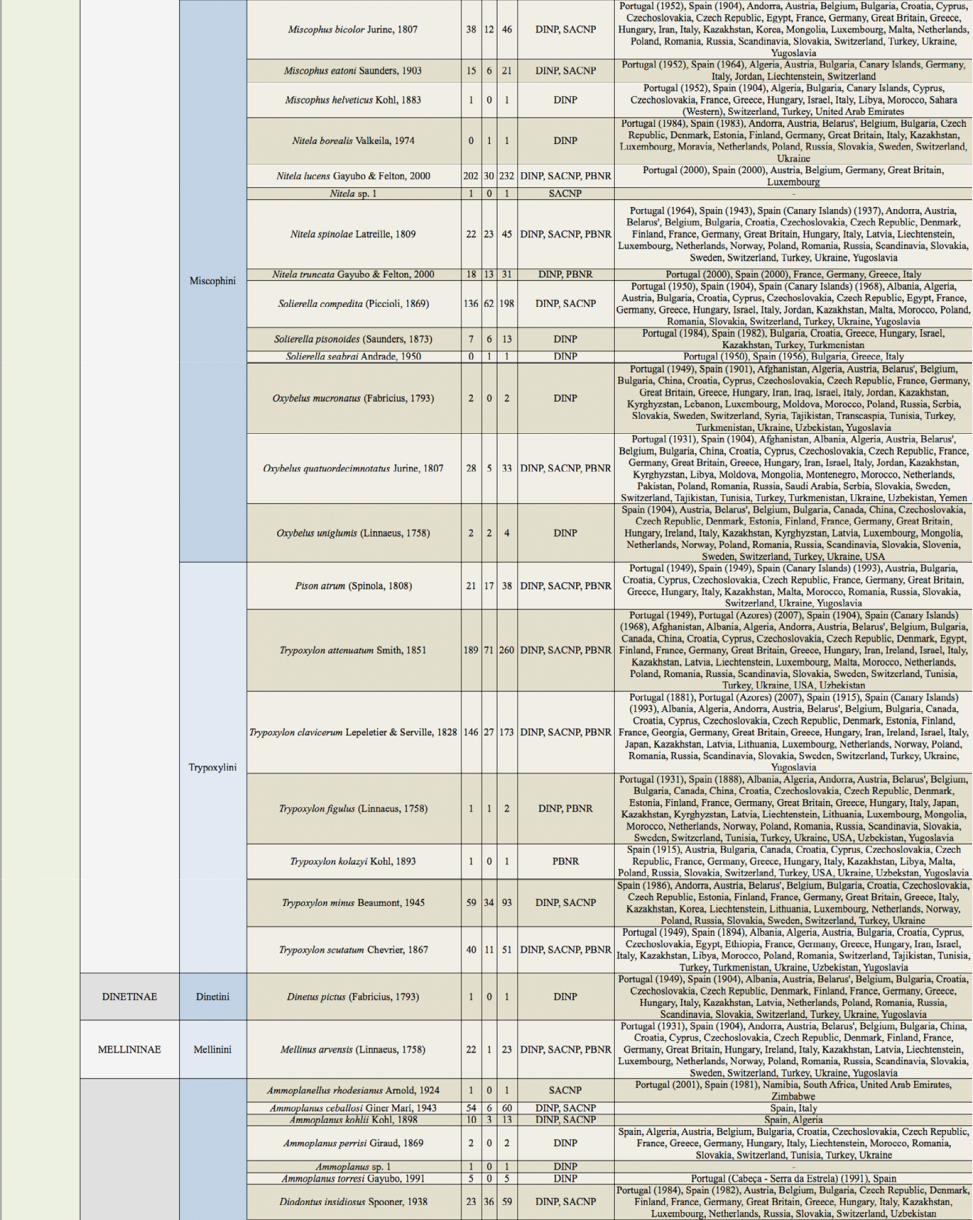

**Continued.**
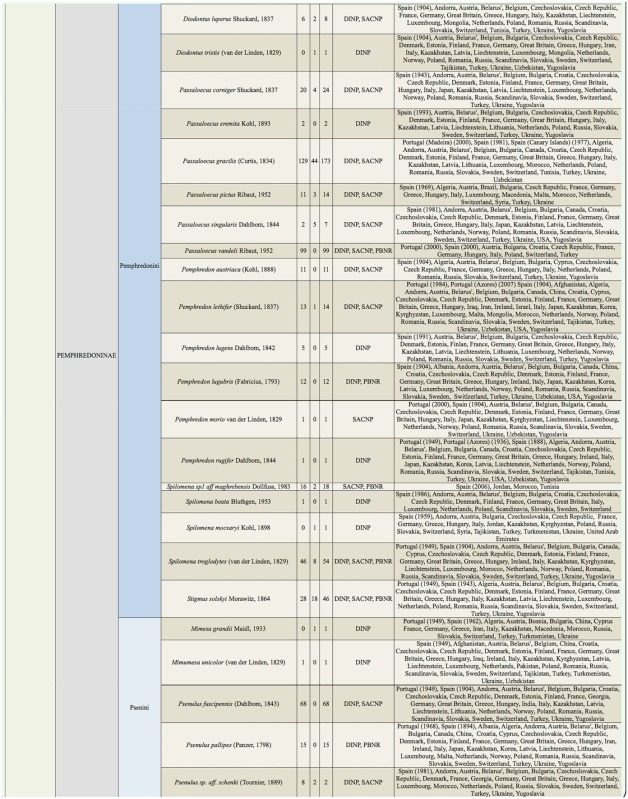

**Continued.**
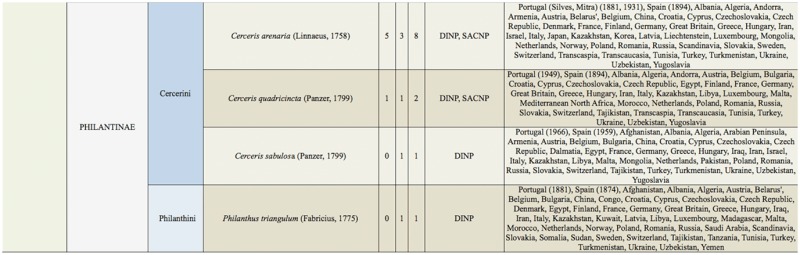

**Supplemental Table 3.**
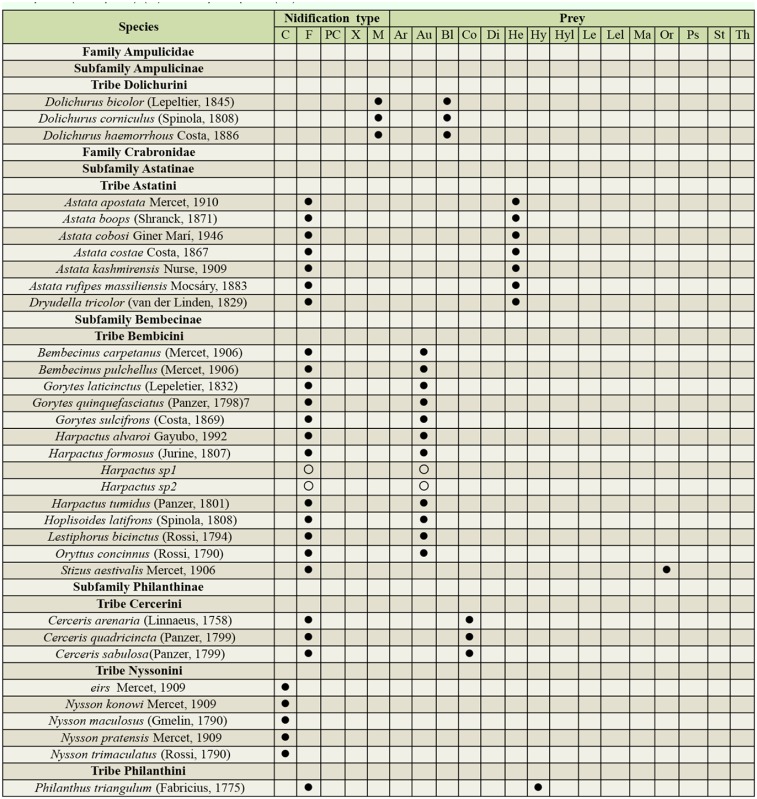
List of taxa collected at the three protected study areas in Portugal showing the nidification type and prey order consumed. (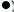) indicates the classification for the species, (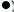
p) indicates the primary prey order when there is more than one, and (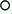) indicate an educated guess of the classification of a morphospecies based on knowledge of the genera. Nidification types: Cleptoparasite (C), Fossorial (F), Pre-existing cavities (PC), Xylicolous (X) and Mixed behavior (M). Prey order: Araneae (Ar), Auchenorrhyncha (Hemiptera) (Au), Blattodea (Bl), Coleoptera (Co), Diptera (Di), Heteroptera (Hemiptera) (He), Hymenoptera (Hy), Hymenoptera larvae (Hyl), Lepidoptera (Le), Lepidoptera larvae (Lel), Mantodea (Ma), Orthoptera (Or), Psocoptera (Ps), Sternorrhyncha (Hemiptera) (St), and Thysanoptera (Th).

**Continued.**
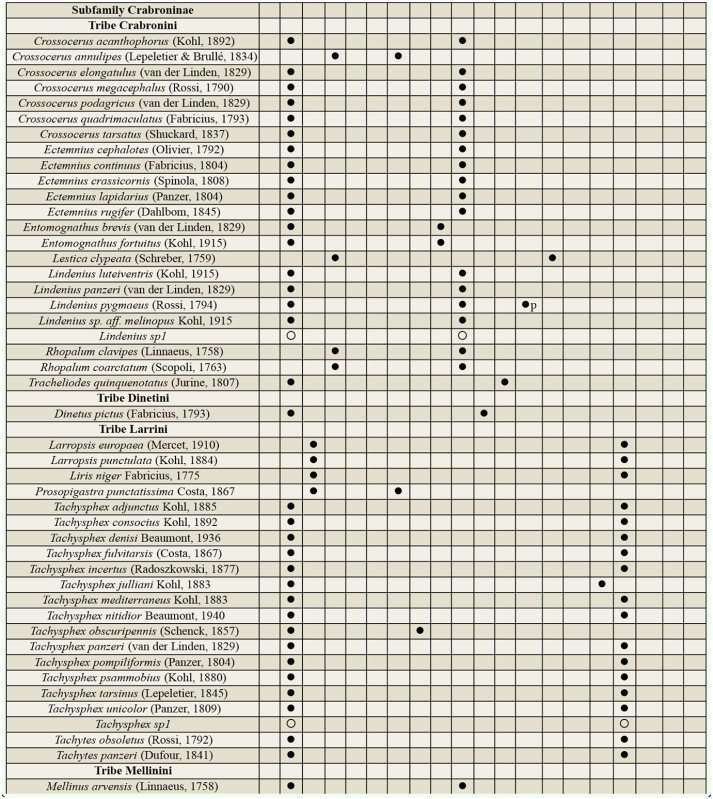

**Continued.**
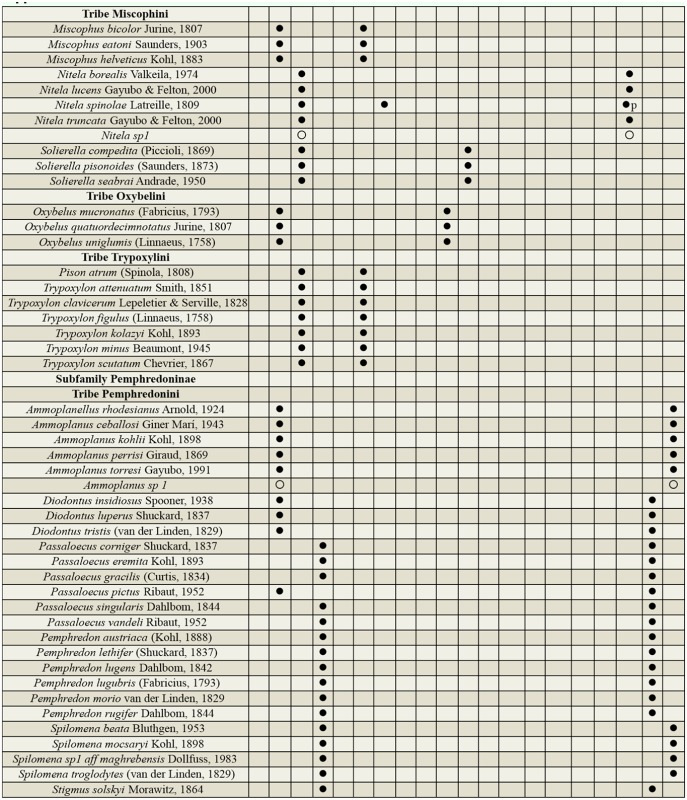

**Continued.**
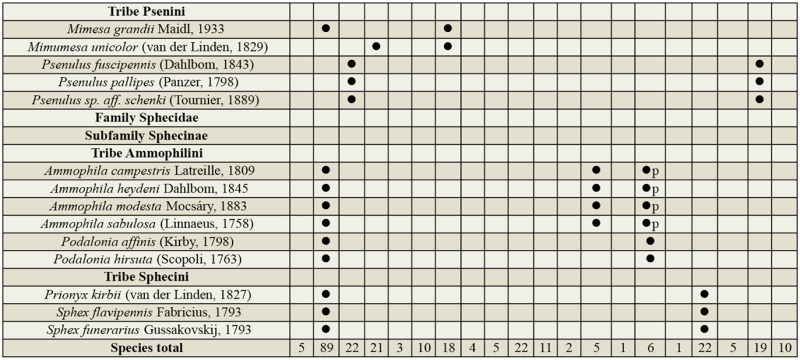

## Abbreviations

DINP: Douro International Natural Park
PBNR: Paúl do Boquilobo Nature Reserve
SACNP: Serras de Aire e Candeeiros Natural Park
